# Long-Term Effects of Cold on Growth, Development and Yield of Narrow-Leaf Lupine May Be Alleviated by Seed Hydropriming or Butenolide

**DOI:** 10.3390/ijms19082416

**Published:** 2018-08-16

**Authors:** Agnieszka Płażek, Franciszek Dubert, Przemysław Kopeć, Michał Dziurka, Agnieszka Kalandyk, Jakub Pastuszak, Piotr Waligórski, Bogdan Wolko

**Affiliations:** 1 Department of Plant Physiology, University of Agriculture, Podłużna 3, 30-239 Kraków, Poland; rrplazek@cyf-kr.edu.pl (A.P.); kubapaaa@gmail.com (J.P.); 2 Polish Academy of Sciences, Institute of Plant Physiology, Niezapominajek 21, 30-239 Kraków, Poland; dubert@ifr-pan.edu.pl (F.D.); przemyslawkopec@gmail.com (P.K.); akalandyk@interia.pl (A.K.); p.waligorski@ifr-pan.edu.pl (P.W.); 3 Polish Academy of Sciences, Institute of Plant Genetics, Strzeszyńska 34, 60-479 Poznań, Poland; bwol@igr.poznan.pl

**Keywords:** amylase, butenolide, cell membrane permeability, chlorophyll *a* fluorescence, cold, dehydrogenase, growth development phases, *Lupinus angustifolius*, narrow-leaf lupine, phytohormones, seed germination

## Abstract

In this article, the effects of cold on the development of *Lupine angustifolius* and the possibility of mitigating it, via seed hydropriming or pre-treatment with butenolide (10^−6^ M–10^−4^ M), are investigated in two cultivars, differing in their ability to germinate at low temperature. Physiological background of plant development after cold stress was investigated in imbibed seeds. For the first four weeks, the seedlings grew at 7 °C or 13 °C. Seeds well germinating at 7 °C demonstrated higher activity of α-amylase and higher levels of gibberellins, IAA and kinetin. Germination ability at low temperature correlated with dehydrogenase activity and membrane permeability. Seed pre-treatment improved germination at low temperature by decreasing abscisic acid content. Seed hydropriming alleviated cold effects on plant development rate and yield, while butenolide accelerated vegetative development but delayed the generative phase. Potential seed yield may be predicted based on the seed germination vigour and the photosynthetic efficiency measured before flowering.

## 1. Introduction

Seed germination consists of several stages, and imbibition is the key stage. The process of imbibition is rapid at optimal temperature. However, when occurring in cold water in soil, it is slow and hazardous for embryos. This is because the cell membranes, which have not adapted to the cold, cannot withstand water pressure, and may rupture [1]. Temperature considerably affects both radicle emergence and seedling growth [2]. Outside optimal temperature range seed germination and seedling establishment decline progressively. Prolonged or temporary exposure to extreme temperatures causes poor seedling development [3]. One way to improve germination at low temperature is seed hydropriming at optimal temperature. Initial hydration of seeds was found to protect cell membranes against cold-induced damage. Dubert and Filek [4] reported that 100% of hydroprimed soybean seeds germinated at 5 °C without any structural or functional damage.

Germination is controlled not only by external factors such as light, temperature or moisture but also by internal growth regulators, especially gibberellins (GAs) and abscisic acid (ABA). Gibberellins are necessary for seed germination as they release coat dormancy, induce endosperm and elongation of the embryo cell. The endosperm is activated by modification of its cell wall proteins [5]. Gibberellins stimulate synthesis and production of hydrolases, especially α-amylase, proteases and β-glucanases [6]. Germination depends on the ratio between GAs and ABA. ABA inhibits embryo expansion and radicle growth, whereas GAs promote these processes [7]. The inhibitory activity of ABA delays the radicle expansion and “weakens” the endosperm, as well as enhances expression of transcription factors that may unfavourably affect the germination [8]. It is generally accepted that ABA inhibits seed germination—but according to Braun and Kahn [9], a decrease in the endogenous level of ABA does not always correlate with germination ability, or is not sufficient to break dormancy in some dormant seeds [10]. Humplik et al. [11] reported that ABA was essential for hypocotyl elongation and that endogenous level of ABA determined growth of tomato seedlings.

Auxins are plant hormones that play a key role in the regulation of cell cycle, growth and development, as well as formation of vascular tissues. The growth of embryos, leaves and roots is regulated by auxin transport. Auxin presence is not required for seed germination, but they are necessary for the growth of young seedlings. These hormones may be found in seed radicle tip during and after germination [8].

Seed germination is also controlled by cytokinins, the receptors of which are responsible of regulating embryo development by affecting the cellular division, seed size, seed production, hypocotyl and shoot growth [12].

Brassinosteroids (BRs) are a class of plant hormones, similar to steroid hormones in other organisms. They exhibit a wide range of activities that induce plant growth and development, seed germination and production of flowers and fruits [13]. Brassinosteroids may enhance seed germination by controlling the inhibitory effects of ABA and enhancing the embryo growth [14].

Karrikins are another group of compounds stimulating seed germination. They are a chemically defined family of plant growth regulators discovered in smoke from burning plant material [15]. Studies by Rokich et al. [16] and Van Staden et al. [17] demonstrated that cold smoke water had a similar effect on seed germination, due to active compounds in the smoke, known as butenolide, and karrikins (KAR), derivatives of butenolide [18]. Butenolide is a heat-stable, long-lasting and water-soluble compound that stimulates seed germination and seedling growth at very low concentrations (in the range of 1 to 100 nM) [19]. Moreover, Jain et al. [3] and Stevens et al. [20] found that butenolide increased seed germination rate as well as seedling vigour of various plant species more effectively than smoke water. In the last decade, butenolide has been used as a stimulator of seed germination and seedling growth of crop plants [18,20,21]. Jain et al. [3] reported that butenolide could overcome temperature stress during tomato seed germination and subsequent growth of the seedlings. Seeds of many plant species, including crop plants originating from various world regions, show a positive response to the compounds of smoke [22].

Methods based on chlorophyll fluorescence are valuable tools for studying the response of photosynthetic apparatus to stress and various environmental factors [23]. Chlorophyll fluorescence can also be used to predict yields of crop plants under diverse environmental conditions [24]. Determination of chlorophyll fluorescence parameters provides information on the efficiency of photosynthetic apparatus by establishing the amount of the energy used in photochemical processes, as well as the energy lost as heat.

In recent years, Polish breeders have been trying to increase the area of *Lupinus* sp. cultivation, as the species have a huge potential of fulfilling protein requirements in animal feed. Special attention has been focused on *L. angustifolius* that has many positive characteristics, such as reliable maturation in the summer, reasonable disease resistance, high yield, and good adaptation to a wide range of environments. Narrow-leaf lupine is a valuable plant species due to its long root system, the ability to absorb nitrogen thanks to symbiotic bacteria of *Bradyrhizobium*, and high protein content [25].

The aim of the study was to investigate the effects of cold temperature (7 °C) during germination on vegetative and generative development of *Lupine angustifolius* and to mitigate them via seed hydropriming or butenolide treatment. The study was performed on a German cultivar Sethes Fruhe Rote and a Polish cultivar Lazur with different ability to germinate at low temperature (7 °C). These cultivars were chosen based on previous experiments [26]. As per our experience, the temperature below 7 °C limits narrow-leaf lupine seed germination, while 13 °C is optimal for germination and growth at early developmental stages. The temperature of 7 °C was the lowest at which the seeds of narrow-leaf lupine were capable of germinating under laboratory conditions.

Physiological and biochemical background of plant development after cold stress was investigated in the imbibed seeds based on such parameters as ion leakage through cell membranes, dehydrogenase and α-amylase activity, and phytohormone content. At later developmental stages, the following parameters were determined: Seedling vigour, fresh and dry weight of shoots, kinetics of chlorophyll *a* fluorescence, vegetative and generative development and yield elements (number of pods and seeds and seed weight per plant).

## 2. Results

### 2.1. Seed Germination Vigour Index

Analysis of variance showed significant differences between the studied cultivars in their ability to germinate at low temperature (Table 1).

**Figure 1:**
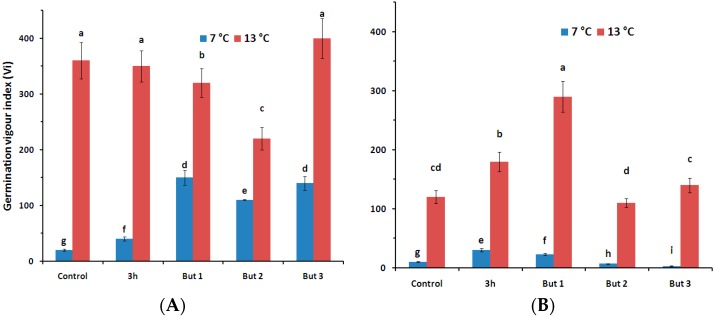
Germination vigour index (Vi) of seeds of *L. angustifolius* cultivars (Lazur—(**A**) and Sethes Fruehe Rote—(**B**)) germinated at 7 °C or 13 °C in water (control), butenolide or hydroprimed in water for 3 h at 20 °C. But 1, But 2, But 3: 10^−6^ M, 10^−5^ M, 10^−4^ M butenolide, respectively. Values represent means (*n* = 5) ± SE. Different letters over error bars (a, b, c, …) indicate significant differences (Duncan’s multiple range test, *p* < 0.05).

Seed germination vigour of both studied cultivars was greater at 13 °C than at 7 °C (Figure 1). Lazur demonstrated much higher values of germination vigour index (Vi) than Sethes Fruehe Rote, which corroborated earlier observations that the latter cultivar is considerably more sensitive to cold. Germination at low temperature was improved by butenolide at all applied concentrations in the case of Lazur seeds, and only at 10^−6^ M (But 1) in Sethes Fruehe Rote. Positive effect of butenolide on the germination vigour at 13 °C was visible for But 3 treatment (10^−4^ M) for Lazur seeds, and for But 1 for Sethes Fruhe Rote. Three-hour hydropriming increased Vi of both cultivars but to a much lower extent than butenolide.

### 2.2. Electrolyte Leakage (EL)

**Figure 2:**
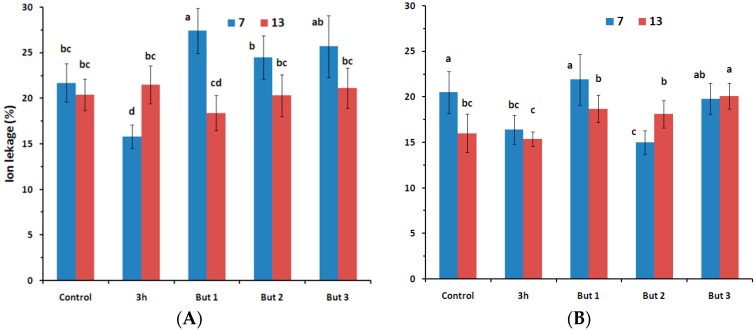
Percentage ion leakage from seeds of two *L. angustifolius* cultivars (Lazur—(**A**); and Sethes Fruehe Rote—(**B**)) germinated at 7 °C (blue bars) or 13 °C (red bars) in water (control), butenolide or hydroprimed in water for 3 h at 20 °C. But 1, But 2, But 3: 10^−6^ M, 10^−5^ M, 10^−4^ M butenolide, respectively. Letters over error bars (±SE; *n* = 10) indicate significant differences (Duncan’s multiple range test, *p* < 0.05).

Cell membrane permeability in seeds depended on cultivar, germination temperature and pre-sowing treatment (Figure 2). Cold increased EL in seeds of both studied cultivars as compared to seeds imbibed at 13 °C, however Lazur seeds seemed more resistant to the cold than those of Sethes Fruehe Rote. At 7 °C, the seeds of both cultivars demonstrated a decline in ion leakage after three-hour hydropriming. In cold-treated Lazur seeds, all concentrations of butenolide increased EL. Contrary to this, Sethes Fruehe Rote seeds showed lower ion leakage under But 1 and But 2 than the controls.

### 2.3. Dehydrogenase Activity (DA)

**Figure 3:**
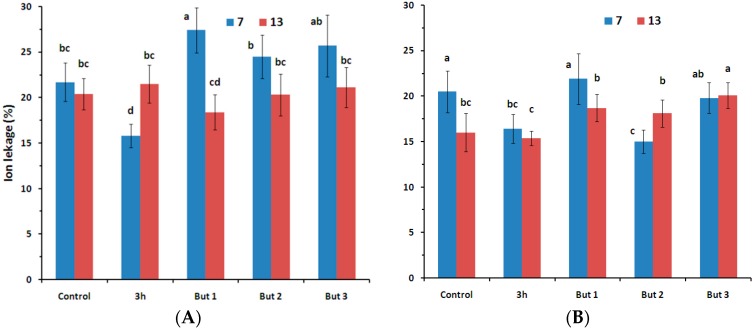
Dehydrogenase activity (DA) [μg formazan·g^−1^ protein·min^−1^] in the seeds of two *L. angustifolius* Lazur cultivars (Lazur—(**A**); and Sethes Fruehe Rote—(**B**)) germinating at 7 °C or 13 °C in water (control), butenolide or hydroprimed in water for 3 h at 20 °C. But 1, But 2, But 3: 10^−6^ M, 10^−5^ M, 10^−4^ M butenolide, respectively. Different letters over error bars (±SE; *n* = 5) indicate significant differences (Duncan’s multiple range test, *p* < 0.05).

Temperature and pre-sowing treatments most strongly affected dehydrogenase activity (Table 1). The seeds of Sethes Fruehe Rote showed considerably lower DA at 7 °C than those of Lazur (Figure 3). The latter did not demonstrate differences in DA under any treatment or temperature, while in the control seeds of Sethes Fruehe Rote the activity of dehydrogenases was higher at 13 °C than at 7 °C. Contrary to all concentrations of butenolide, 3 h hydropriming did not enhance DA in the seeds of any cultivar at any temperature. The highest DA was noticed at 13 °C in the Lazur seeds germinating under But 2 treatment and in those of Sethes Fruehe Rote treated with But 1 and But 3.

### 2.4. α-Amylase Activity (A)

**Figure 4:**
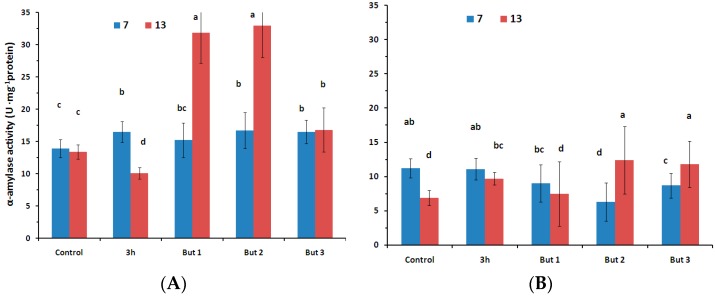
Activity of α-amylase [U·mg^−1^ protein] in the seeds of two *L. angustifolius* cultivars (Lazur—(**A**); and Sethes Fruehe Rote—(**B**)) germinating at 7 °C or 13 °C in water (control), butenolide or hydroprimed in water for 3 h at 20 °C. But 1, But 2, But 3: 10^−6^ M, 10^−5^ M, 10^−4^ M butenolide, respectively. Different letters over error bars (±SE; *n* = 3) indicate significant differences (Duncan’s multiple range test, *p* < 0.05).

The analysis of variance showed the influence of cultivar, temperature and seed treatments on the activity of α-amylase in the germinating seeds (Table 1). The enzyme activity in both studied cultivars was generally low at 7 °C and 13 °C (Figure 4), but it was higher in Lazur seeds. Three-hour hydropriming enhanced α-amylase activity in Lazur seeds at 7 °C and in Sethes Fruehe Rote seeds at 13 °C. Butenolide in all used concentrations stimulated the enzyme in Lazur seeds at both temperatures and only at 13 °C in Sethes Fruehe Rote seeds.

### 2.5. Hormone Content

Temperature and pre-sowing treatments significantly affected hormone content in imbibed seeds of the studied cultivars (Table 1). The experiment confirmed the presence of active gibberellins (GA_1_, GA_3_, GA_4_, GA_5_, GA_6_, collectively GAs), and inactive GA_8_ (one of products of gibberellins deactivation).

**Figure 5:**
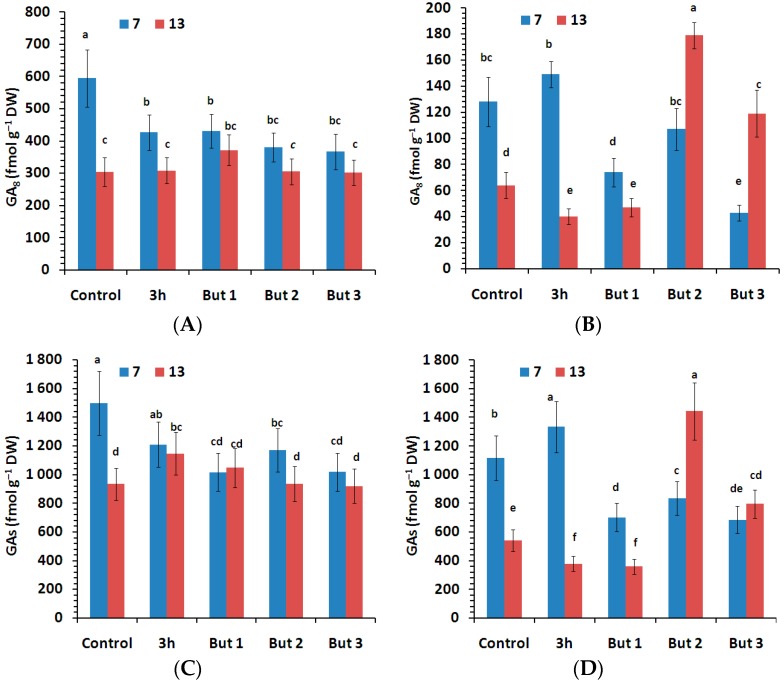
Content of non-active form of gibberellins (GA_8_) and sum of active gibberellins (GA_1_, GA_3_, GA_4_, GA_5_, GA_6_) measured in the seeds of two *L. angustifolius* cultivars (Lazur—(**A**,**C**); Sethes Fruehe Rote—(**B**,**D**)) germinated at 7 °C or 13 °C in water (control), butenolide or hydroprimed in water for 3 h at 20 °C. But 1, But 2, But 3: 10^−6^ M, 10^−5^ M, 10^−4^ M butenolide concentrations. Different letters over error bars (±SE; *n* = 3) indicate significant differences (Duncan’s multiple range test, *p* < 0.05).

The content of active gibberellins was given as their total amount (Figure 5). In the control seeds of Sethes Fruehe Rote, the amount of GA_8_ and active GAs was considerably lower than in Lazur ones. Generally, the level of GA_8_ was higher in the seeds of both cultivars imbibed at 7 °C than at 13 °C, with an exception of Sethes Fruehe Rote seeds germinating under But 2 and But 3 treatments. Three-hour hydropriming increased the level of this hormone in Sethes Fruehe Rote seeds at 7 °C as compared with that of the control. Butenolide in all concentrations significantly reduced the content of GA_8_ in the seeds of both cultivars at 7 °C. Similarly to GA_8_, the levels of active GAs were higher at 7 °C than at 13 °C. In Sethes Fruehe Rote seeds only 3-h hydropriming increased GAs levels at low temperature as compared to the control. In Lazur cultivar, the highest content of GAs was observed in the control seeds at 7 °C.

**Figure 6:**
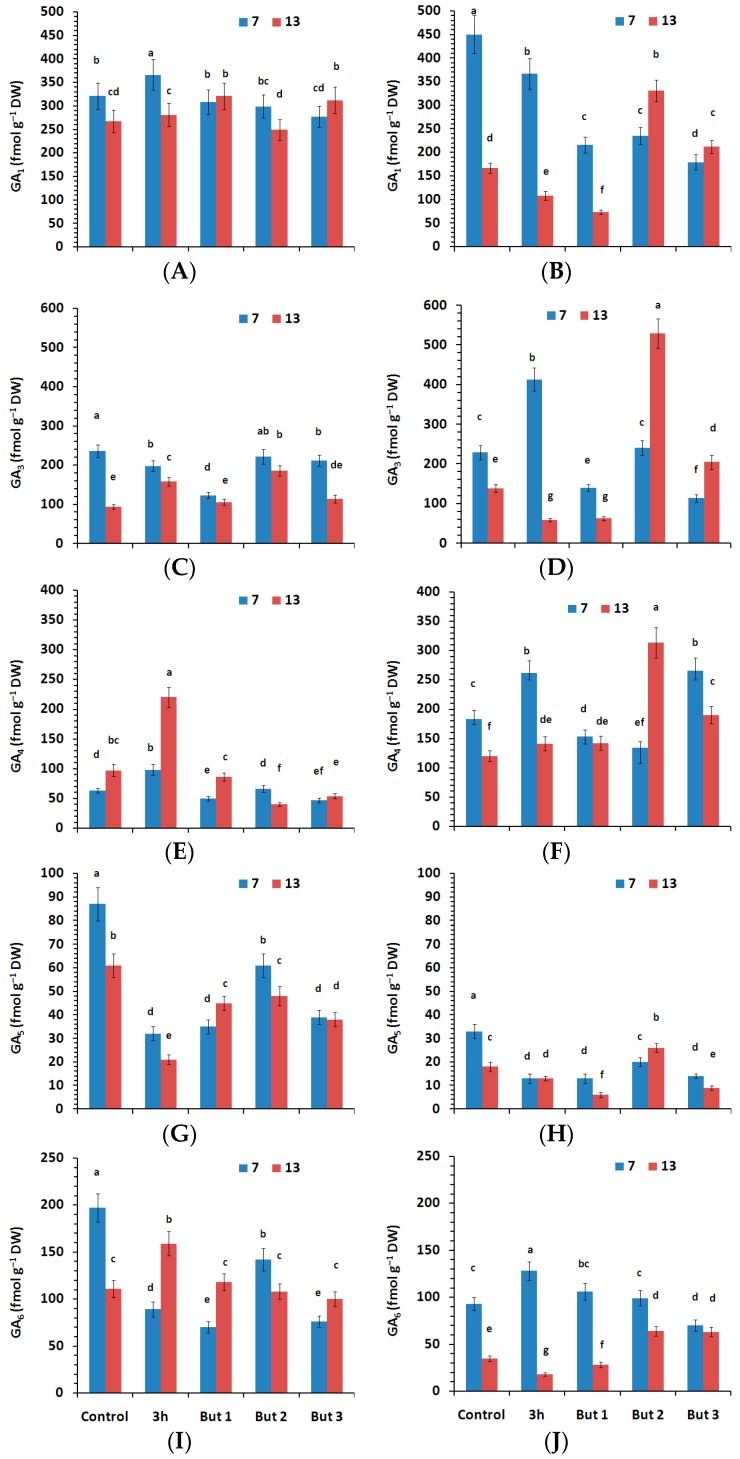
Content of active gibberellins (GA_1_, GA_3_, GA_4_, GA_5_, GA_6_) measured in the seeds of two *L. angustifolius* cultivars (Lazur—(**A**,**C**,**E**,**G**,**I**); Sethes Fruehe Rote—(**B**,**D**,**F**,**H**,**J**)) germinated at 7 °C or 13 °C in water (control), butenolide or hydroprimed in water for 3 h at 20 °C. But 1, But 2, But 3: 10^−6^ M, 10^−5^ M, 10^−4^ M butenolide concentrations. Different letters over error bars (±SE; *n* = 3) indicate significant differences (Duncan’s multiple range test, *p* < 0.05).

Gibberellin GA_5_ was the least abundant of the other active gibberellins (Figure 6). Lazur control seeds at 7 °C showed higher content of GA_3_, GA_5_ and GA_6_ than Sethes Fruehe Rote seeds, and lower levels of GA_1_ and GA_4_. At 13 °C Lazur seeds contained considerably more GA_1_, GA_5_ and GA_6_ than Sethes Fruehe Rote ones. Only hydropriming enhanced the levels of GA_1_ and GA_4_ in Lazur seeds germinating at 7 °C. In Sethes Fruehe Rote seeds this effect at the same temperature was observed for GA_3_ and GA_4_ following hydropriming, and in GA_4_ after But 2 and But 3 treatments. An increase in active gibberellin content in Lazur seeds at 13 °C was observed after hydropriming (GA_1_, GA_3_, GA_4_, GA_6_) and butenolide treatment (GA_1_ and GA_3_). In Sethes Fruehe Rote seeds at 13 °C butenolide increased the levels of all studied active gibberellins.

**Figure 7:**
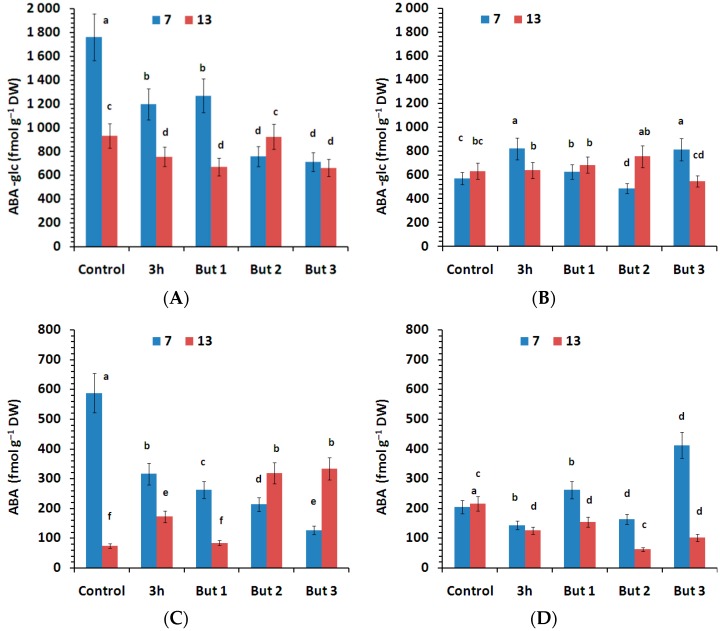
Content of inactivated form of ABA (ABA-glc) and active ABA measured in the seeds of two *L. angustifolius* cultivars (Lazur—(**A**,**C**); Sethes Fruehe Rote—(**B**,**D**)) germinated at 7 °C or 13 °C in water (control), butenolide or hydroprimed in water for 3 h at 20 °C. But 1, But 2, But 3: 10^−6^ M, 10^−5^ M, 10^−4^ M butenolide concentrations. Different letters over error bars (±SE; *n* = 3) indicate significant differences (Duncan’s multiple range test, *p* < 0.05).

Abscisic acid was detected in an inactive, conjugated form with glucose (ABA-glc), and in the free form. Lazur seeds showed higher level of ABA-glc than the seeds of Sethes Fruehe Rote (Figure 7). Lazur seeds had also higher content of ABA-glc at 7 °C than at 13 °C. In Sethes Fruehe Rote seeds, the level of this ABA form depended on the interaction between temperature and seed treatment, especially after 3 h-hydropriming and under But 3 treatment. In Lazur seeds, all applied treatments depleted the inactive form of ABA. The seeds of Sethes Fruehe Rote showed lower levels of free ABA as compared to Lazur. Hydropriming and But 2 decreased ABA content in Sethes Fruehe Rote seeds germinating at 7 °C as compared to that of the control, while in Lazur seeds all treatments reduced ABA level.

**Figure 8:**
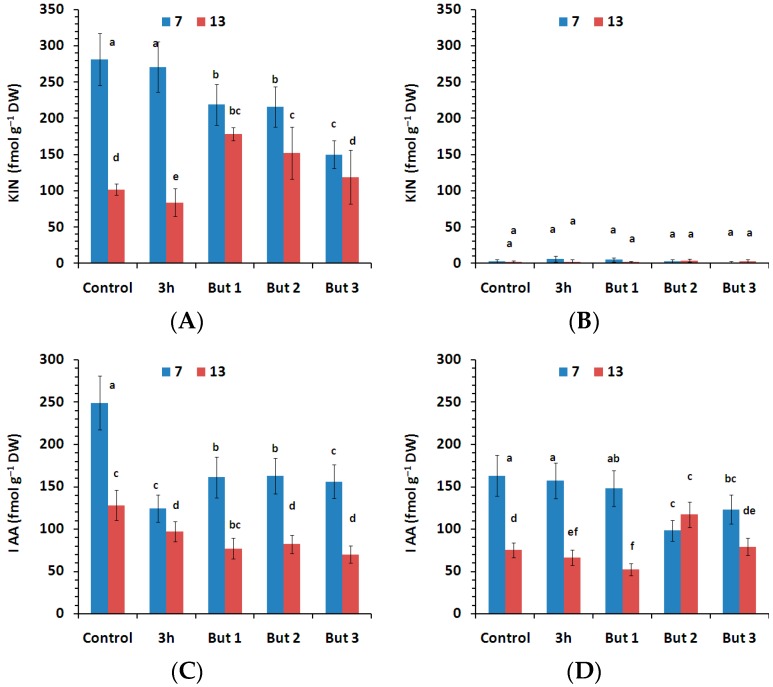
Content of KIN—kinetin abd IAA—indolile-3-acetic acid measured in the seeds of two *L. angustifolius* cultivars (Lazur—(**A**,**C**); Sethes Fruehe Rote—(**B**,**D**)) germinated at 7 °C or 13 °C in water (control), butenolide or hydroprimed in water for 3 h at 20 °C. But 1, But 2, But 3: 10^−6^ M, 10^−5^ M, 10^−4^ M butenolide concentrations. Different letters over error bars (±SE; *n* = 3) indicate significant differences (Duncan’s multiple range test, *p* < 0.05).

Sethes Fruehe Rote seeds demonstrated markedly lower level of (kinetin) KIN in all variants as compared with Lazur (Figure 8A,B). The amount of this hormone most strongly differentiated the investigated cultivars. In Lazur seeds the amount of KIN was higher at 7 °C than at 13 °C. The stimulating factors did not affect KIN level in the seeds of any cultivar as compared with controls. Other cytokinins, i.e., *cis*- and *trans*-zeatin, their respective rybosides as well as isopentenyladenine, were also determined. However, their amount was very low (3–7 fmol·g^−1^ dry weight (DW)) regardless of treatment and cultivar.

Lazur seeds showed higher content of IAA than Sethes Fruehe Rote ones (Figure 8c,d). This hormone was more abundant in the seeds germinating at 7 °C than at 13 °C. In both studied cultivars neither hydropriming nor butenolide increased IAA levels in the seeds.

**Figure 9:**
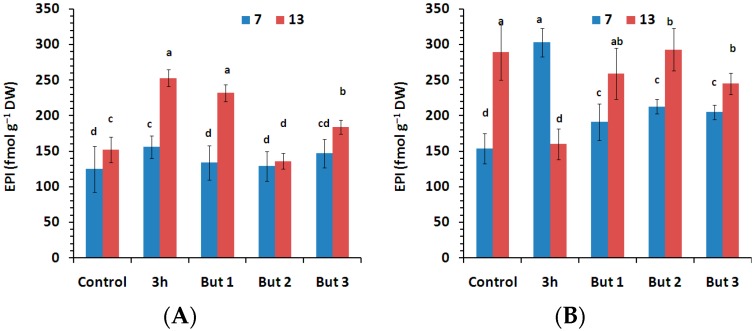
Content of EPI—24-epibrassinolide measured in the seeds of two *L. angustifolius* cultivars (Lazur—(**A**); Sethes Fruehe Rote—(**B**)) germinated at 7 °C or 13 °C in water (control), butenolide or hydroprimed in water for 3 h at 20 °C. But 1, But 2, But 3: 10^−6^ M, 10^−5^ M, 10^−4^ M butenolide concentrations. Different letters over error bars (±SE; *n* = 3) indicate significant differences (Duncan’s multiple range test, *p* < 0.05).

The content of 24-epibrassinolide (EPI) was higher in the seeds of Sethes Fruehe Rote than in Lazur, and in both cultivars the content of this hormone was higher at 13 °C than at 7 °C (Figure 9). The stimulating effect of hydropriming and butenolide on EPI levels was observed mainly in Sethes Fruehe Rote seeds at 7 °C. The highest amount of this hormone was found in the seeds of this cultivar after 3-h hydropriming.

### 2.6. Vegetative Phase Development (VPD)

Vegetative development of plants grown for the first four weeks at two different temperatures depended on cultivar and pre-sowing treatments (Table 2).

In both cultivars, index of VPD was lower at 7 °C than at 13 °C, however, in Lazur it was higher than in Sethes Fruehe Rote (Table 3). At 7 °C, butenolide in all concentrations decreased VPDi of Lazur plants as compared to that of the control. Development of Sethes Fruehe Rote plants growing in the beginning of the experiment at 7 °C was weaker under But 1 and But 2 treatments, while 3-h hydropriming and the highest butenolide concentration (But 3) accelerated their vegetative growth. At 13 °C, no pre-sowing treatments affected VPDi of Lazur plants. In Sethes Fruehe Rote the seed hydropriming had no effect on this parameter but butenolide in all used concentrations improved VPDi.

### 2.7. Fresh (FW) and Dry Weight (DW)

Statistical analysis showed that FW and DW of shoots were strongly influenced by growth temperature, and to a smaller degree by pre-sowing treatments (Table 2). The effects of low temperature applied during the first four weeks persisted long time after the temperature was increased. All plants cultivated at 7 °C in the beginning of the experiment developed smaller leaves and were shorter than those grown at 13 °C but they had a more compact habit. Control plants of both cultivars showed greater shoot FW and DW at 13 °C than at 7 °C (Table 4).

Seed hydropriming and But 2 increased FW and DW of Lazur plants grown for the first four weeks at both temperatures. Sethes Fruehe Rote plants did not respond by weight gain to any seed treatment irrespective of cultivation temperature.

### 2.8. Chlorophyll a Fluorescence

**Figure 10:**
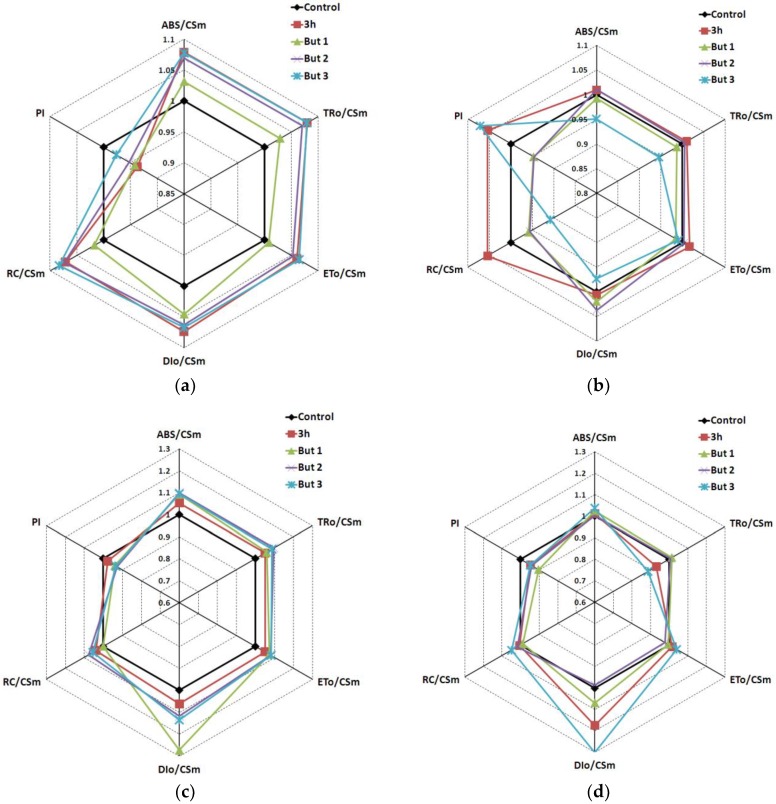
Radar plot of chlorophyll *a* fluorescence kinetics parameters of two *L. angustifolius* cultivars germinated and grown for the first four weeks at 7 °C (Lazur—(**a**); Sethes Fruehe Rote—(**b**)) or 13 °C (Lazur—(**c**); Sethes Fruehe Rote—(**d**)). Control—plants grown from seeds germinated in water; 3 h—plants grown from seeds hydroprimed for 3 h at 20 °C; But 1, But 2, But 3—plants grown form seeds germinated in the presence of 10^−6^, 10^−5^ and 10^−4^ M butenolide, respectively. Black line indicates to control plants as 100%. Values represent means (*n* = 10).

The kinetics of chlorophyll *a* fluorescence, with an exception of the number of active reaction centres (RC/CSm), varied mainly between cultivars and growth temperature during the first weeks of cultivation (Table 5). Light energy absorption by antenna of PSII (ABS/CSm) in control plants of both studied cultivars was lower at 13 °C than at 7 °C (Figure 10). In Lazur plants, all pre-sowing treatments increased ABS/CSm at both temperatures, while in Sethes Fruehe Rote an increase in this parameter was observed only at 13 °C after seed treatment with But 1 and But 2.

Similarly to energy absorption by PSII antennas, the amount of excitation energy trapped in PSII (TRo/CSm) in the leaves of both cultivars was higher in plants grown at 7 °C than at 13 °C (Figure 10). In Lazur plants grown at both temperatures, TRo/CSm increased after 3-h hydropriming and treatment with butenolide in all concentrations. In Sethes Fruehe Rote plants cultivated for the first weeks at 7 °C, But 1 and But 3 reduced the value of TRo/CSm. This attribute increased at 13 °C as a result of seed hydropriming and But 3 treatment. 

The amount of energy used for electron transport (ETo/CSm) determined in the leaves of cv. Lazur was similar in plants grown in the beginning at both temperatures and it increased in response to all seed treatments (Figure 10). In Sethes Fruehe Rote ETo/CSm was significantly higher in plants transferred to the open foil tunnel from 13 °C than from 7 °C. Pre-sowing treatments did not affect this parameter.

Dissipation of energy from PSII (DIo/CSm) was greater in control plants of both cultivars growing at 7 °C than at 13 °C (Figure 10). All pre-sowing treatments of Lazur seeds enhanced the energy dissipation at both temperatures. In the case of cv. Sethes Fruehe Rote, this effect was recorded only in plants cultivated at 13 °C after 3 h, But 1 and But 3 treatments.

The number of active reaction centres (RC/CSm) in all plants under study did not depend on the cultivar or growth temperature (Table 5). The values of RC/CSm increased in Lazur plants grown at 7 °C and 13 °C from hydroprimed seeds and after application of butenolide at higher concentrations (But 2 and But 3) (Figure 10). Only 3-h hydropriming increased RC/CSm in Sethes Fruehe Rote plants grown at 7 °C.

The cultivar, growth temperature and seed treatments significantly affected PI values (Table 5), which were higher at the higher temperature (Figure 10). Positive effects of pre-sowing treatments on PI were visible only in Sethes Fruehe Rote plants grown in the beginning of the experiment at 7 °C after seed hydropriming and But 3 treatment. In all other cases, the pre-sowing treatments decreased PI values.

### 2.9. Plant Generative Development (GD)

Generative development proceeded differently in the studied cultivars and depended on temperature applied during the first weeks of growth and pre-sowing treatments (Table 2). In both cultivars, cold inhibited the generative development, which was more visible in cv. Sethes Fruhe Rote (Table 6). It should be remembered that the cultivars differed significantly in their flowering season, which is mainly due to their genetic background. Sethes Fruehe Rote plants were only at the beginning of the generative phase, when most Lazur plants had already developed inflorescences. In both cultivars, only seed hydropriming accelerated the generative development of plants grown for the first four weeks at 7 °C. Seed treatment with butenolide delayed the flowering process, particularly in Sethes Fruehe Rote. In this cultivar, 10^−4^ M butenolide markedly inhibited flowering of plants grown at both 7 °C and 13 °C.

### 2.10. Yield Parameters

Yield was estimated four months after seed sowing. The cultivars differed considerably in pod and seed production (Table 7 and Table 8). Temperatures applied for the first four weeks of growth did not affect all yield parameters of control plants in both studied cultivars. Higher number of pods and seeds per plant regardless of temperature and pre-sowing treatment was recorded in cv. Lazur, while the seeds of Sethes Fruehe Rote were heavier. Lazur plants were more sensitive to stimulating effects of pre-sowing treatments than Sethes Fruehe Rote ones. Butenolide decreased the number of pods in Lazur plants grown at 7 °C, while at 13 °C only But 1 evoked this effect. Butenolide reduced the number of pods in Sethes Fruehe Rote plants grown at 13 °C, while seed hydropriming enhanced pod number in both cultivars.

In Lazur plants grown in the beginning at 7 °C, seed hydropriming and But 2 increased the seed number over control (Table 8). In plants of this cultivar maintained at 13 °C, the same treatments and But 3 increased the yield. Seed hydropriming significantly enhanced seed yield in both cultivars. It is worth adding that butenolide, especially in higher concentrations applied during germination, reduced seed yield of Sethes Fruehe Rote plants. In Lazur plants grown at 7 °C, But 1 and But 3 declined the seed number, and at 13 °C this effect was noted after seed treatment only with But 1. Similar result was obtained for seed weight (Table 8). It was stimulated in both cultivars most visibly after seed hydropriming, while butenolide at 10^−5^ M increased Lazur seed weight at both temperatures. Reassuming, seed hydropriming was the most effective in enhancing all yield parameters, while butenolide showed both stimulatory and inhibitory effects.

### 2.11. Correlations Between Investigated Parameters

The study outcomes were analysed for possible correlations between the examined parameters. All presented Pearson’s coefficients of correlation were significant at *p* < 0.05. The most interesting results showed a relationship between the germination vigour index (Vi) and the following parameters: The number of pods (*r* = 0.348), fresh (*r* = 0.502) and dry weight (*r* = 0.509) of plants, overall efficiency of photosynthesis (PI) (*r* = 0.449) and energy transported outside plastoquinone Q_A_ (ETo/CSm) (*r* = 0.400). Another interesting relationship was found between the number of pods and seeds and such hormones as inactive GA_8_ (*r* = 0.300 and *r* = 0.334, respectively) and KIN (*r* = 0.483 and *r* = 0.367, respectively) determined in the seeds one day after sowing. In addition, FW of the shoots significantly affected the number of pods and seeds (*r* = 0.312 and *r* = 0.337, respectively) and correlated with fluorescence parameters such as PI (*r* = 0.646) and ETo/CSm (*r* = 0.365).

## 3. Discussion

The influence of cold on plant development has been very well documented by many authors [1,2,3,26,27]. Seed germination is a complex process, regulated by various endogenous and external factors. One of them is temperature that plays a very important role during seed germination, radicle emergence and post-germinative growth of seedlings [3]. Our study demonstrated that temperature during germination and the first weeks of seedling growth could affect vegetative and generative plant development. The experiment involved two cultivars of narrow-leaf lupine differing in their ability to germinate at low temperature. Cultivar Lazur was recognised as more resistant to cold than Sethes Fruehe Rote. Although the seeds of both cultivars germinated slower at 7 °C than at 13 °C, the seed germination vigour index (Vi) of cv. Lazur was higher at low temperature than of cv. Sethes Fruehe Rote. Germination vigour of both cultivars was improved by butenolide, however, Lazur seeds germinated better in all applied concentrations, while in Sethes Fruehe Rote seeds germination was enhanced only by 10^−6^ M butenolide. Jain et al. [3] showed a stimulating effect of 10^−7^ M butenolide on the germination of tomato seeds below and above optimum temperatures. Stevens et al. [20] reported a stimulating effect of butenolide applied in the field and laboratory conditions on seed germination of species belonging to Brassicaceae, Poaceae and Asteraceae families. These authors demonstrated that maximum germination percentage of various plant species occurred at butenolide concentrations ranging from 0.67 nM to 67 nM. Contrary to that, Brown and Van Staden [28] claimed that smoke extracts at high concentrations might inhibit seed germination. Different concentrations of butenolide may also affect growth of different plant organs. Kulkarni et al. [21] observed that 10^−8^ M butenolide increased length of rice shoots, whereas lower concentration (10^−10^ M) promoted maximum root length and seedling weight. Seed hydropriming also enhanced seed germination but in the studied cultivars this effect was not as visible as after butenolide application. Low temperature increased cell membrane permeability in all plants that declined in 3-h hydroprimed seeds. In Lazur seeds butenolide increased EL, while in Sethes Fruehe Rote ones it triggered an opposite effect. The results of our experiment corroborated the findings of other authors, who reported a negative correlation between seed germination vigour and cell membrane permeability and a positive one with dehydrogenase activity in seeds [29,30]. Cold sensitive Sethes Fruehe Rote demonstrated lower DA at 7 °C, while in the seeds of cold resistant Lazur dehydrogenase activity was similar at 7 °C and 13 °C. Contrary to hydropriming, butenolide enhanced DA in the seeds of both cultivars. Activity of α-amylase in the seeds of both cultivars was relatively low. This effect could be explained by the content of storage materials in lupine seeds. Seeds of this type contain more proteins than starch, so amylolytic activity could be considerably lower as compared with that of grains. In addition, α-amylase activity was higher in Lazur seeds than in Sethes Fruehe Rote. It was enhanced by 3-h hydropriming and butenolide at different concentrations.

Hormone profile of imbibing seeds was also determined. The amount of inactive GA_8_ and active GAs was higher in all seeds imbibed at 7 °C than at 13 °C. The seeds of cold resistant Lazur demonstrated higher level of GA_8_ and GAs than those of Sethes Fruehe Rote, which may prove the key role of not only the amount of these hormones in germinating seeds but also of the ratio of their active and inactive forms [31]. Moreover, it was unusual that the seeds of cold resistant Lazur contained higher levels of GA_3_, GA_5_ and GA_6_ than the seeds of cold sensitive Sethes Fruehe Rote, while the latter produced more GA_1_ and GA_4_. These results indicated that germination at unfavourable temperature might be determined not by the total amount of gibberellins but by the levels of individual gibberellins. Interestingly, Chien et al. [32] reported that a combination of GA_4_ and GA_7_ had a greater effect on the germination of dormant Taxus mairei seeds than GA_4_ alone. Universal occurrence of GA_1_ and GA_4_ in plants suggests that they are functionally active forms and co-occur with their biosynthetic precursors and metabolites, which are often present at much higher concentrations than the hormones themselves [33].

Stimulating effect of the pre-sowing treatments on germination process could be explained by the fact that butenolide reduced the levels of GA_8_, while hydropriming increased the amount of active gibberellins. Zhou et al. [19] reported that butenolide enhanced the expression of two GAs biosynthesis genes (GA3ox1 and GA3ox2) during seed imbibition. Furthermore, Meng et al. [34] demonstrated that karrikins delayed soybean seed germination through enhancing ABA biosynthesis, while impairing GAs biogenesis.

The seeds of cv. Lazur showed higher level of glc-conjugated ABA than those of Sethes Fruehe Rote. This suggested more abundant presence of conjugated ABA in seeds at the lower temperature. The content of this inactive form was reduced by all applied treatments only in Lazur seeds. The seeds of Sethes Fruehe Rote showed lower amount of free ABA, which seemed unexpected considering their lower germination ability at low temperature. It is possible that germination is induced not by absolute levels of ABA but by the ratio of conjugated and free forms of this hormone. This ratio was similar in the seeds of both studied cultivars. According to Braun and Khan [9], a decrease in the endogenous level of ABA does not always correlate with germination ability. Humplik et al. [11] showed that ABA was essential for hypocotyl elongation and that appropriate control of the endogenous ABA level was required to drive the growth of etiolated seedlings. Our results may suggest that the stimulating effect of treatments on seed germination was manifested by reducing the content of free ABA.

Similarly to ABA, Lazur seeds showed higher amount of IAA and KIN than Sethes Fruehe Rote. The IAA also occurred in higher levels in the seeds germinating at 7 °C than at 13 °C but in neither cultivar its amount was affected by seed treatment. While other cytokinines were at low levels an unexpected finding was a considerable difference in the seed content of KIN. The seeds of Sethes Fruehe Rote, weakly germinating at low temperature, showed even order of magnitude lower level of KIN than the well germinating Lazur seeds. This stays in agreement with the key role of cytokinins in cellular division and hypocotyl and shoot growth [12]. Unlike the other hormones, BRs seemed to not affect the germination of narrow-leaf lupine at low temperature.

In our experiment, cold affected plant growth long after their transfer to the higher temperature. The ability to germinate at low temperature correlated with other growth and development parameters. Cold occurring during the initial phase of plant growth decreased fresh and dry weight of shoots. Although both cultivars grew and developed slower at 7 °C, Sethes Fruehe Rote plants, as the ones more sensitive to cold, progressed much slower and were delayed in their vegetative development as compared with Lazur cultivar. It is worth mentioning that contrary to Sethes Fruehe Rote, cold did not delay flowering of Lazur plants. Seed hydropriming was more effective in alleviating the consequences of cold on plant development rate and yield than butenolide. Sethes Fruehe Rote plants were more sensitive to butenolide, which could be explained by fact that the stimulating effects of seed treatments were more visible in more stress-sensitive cultivar. There are very few works on post-germination effects of smoke water or butenolide [35]. Baxter and Van Staden [36] reported growth stimulation in seedlings that sprouted from smoke-treated seeds of *Themeda triandra*. A similar effect was observed by Brown [37] in *Erica* and *Asteraceae* species. Butenolide exerted different effects on the investigated developmental parameters, and it was difficult to state which butenolide concentration evoked the most desired outcomes. Although the stimulating activity of different butenolide concentrations was reported also by other authors, its higher levels triggered rather inhibiting effects [20,21,28]. In our experiment, the effects of butenolide depended on growth temperature and cultivar. Butenolide at 10^−5^ M improved the yield of Lazur plants at 7 °C and 13 °C, while Sethes Fruehe Rote plants showed lower yield after seed treatment with butenolide. Butenolide at 10^−6^ M butenolide accelerated vegetative development of Sethes Fruehe Rote plants grown at 7 °C for the first 4 weeks, whereas in the plants grown at 13 °C, all used butenolide concentrations evoked this effect. Contrary to that, butenolide delayed the generative phase in both cultivars.

Many earlier works involved seed treatment with plant-derived smoke or smoke water (SW), while in this study a single compound butenolide was used. Our previous experiments [26] were carried out on seeds treated with commercial SW and their results indicated that smoke water as a complex mixture of various compounds evoked more pronounced effects than butenolide alone. This could be the reason for different effect of SW on seed germination compared to butenolide. The conclusion is consistent with the results published by Soós et al. [38] and Zhou et al. [19]. Soós et al. [38] found that gene expression and protein ubiquitination patterns in maize kernels treated with SW or KAR1 were quite different.

In our opinion, the most important results of this experiment were significant relationships found between the studied parameters. Seed germination vigour correlated with the number of pods, FW and DW of plants, the overall efficiency of photosynthesis (PI) and a stream of energy transported outside plastoquinone Q_A_ (ETo/CSm). In addition, the level of inactive form of gibberellins (GA_8_) and kinetin in germinating seeds influenced yield parameters. Furthermore, fresh weight of shoots significantly affected seed yield and correlated with chlorophyll *a* fluorescence parameters such as PI and ETo/CSm. This is even more interesting as the measurement date of seed germination vigour (four days after sowing) considerably preceded the date of the remaining analyses (by over four months for yield analysis and by 2.5 months for chlorophyll fluorescence analysis). It should therefore be assumed that potential yield might be predicted based on the seed germination vigour. According to Kalaji and Pietkiewicz [24], the analysis of chlorophyll fluorescence provides a quick insight into photochemical efficiency of plants cultivated under different field conditions. Kalaji et al. [39] used the parameters of chlorophyll *a* fluorescence to predict barley yield seven days after salt stress application, while in our study the distance between stress occurrence and determination of the PI and yield was much longer. The overall efficiency of photosynthesis was improved only in Sethes Fruehe Rote plants grown from seeds treated with the lowest butenolide concentration and germinated at 7 °C.

## 4. Materials and Methods

### 4.1. Plant Material

Seeds of two *Lupinus angustifolius* cultivars: Sethes Fruhe Rote and Lazur were obtained from the collection of the Smolice Plant Breeding Station, Przebędowo, IHAR group, Poland. The seeds were collected in autumn 2016, and storred at 10–15 °C ~30% relative humidity until sowing. The experiments were performed between March and July 2017 in laboratory conditions and in an open foil tunnel.

### 4.2. Experimental Treatments

The seeds were surface sterilised with 70% ethanol and washed three times with sterile water. Plastic Petri dishes (9 cm) containing filter paper were filled with 15 cm^3^ of distilled water (for control and 3-h hydroprimed seeds) or butenolide i.e., 2(5*H*)-furanone) (Sigma-Aldrich, St. Louis, MO, USA) in the following dilutions in distilled water: 10^−6^ M, 10^−5^ M and 10^−4^ M. Seed treatments with the mentioned butenolide concentrations were named But 1, But 2 and But 3, respectively. One hundred fifty seeds of each cultivar were placed in the dishes, fifteen seeds per dish, ten dishes for each combination of cultivar/temperature/treatment. The seeds germinated in dark in phytotronic chambers (phytotron facilities of the Department of Plant Physiology, University of Agriculture in Krakow, Kraków, Poland) at 7 °C (cold) or 13 °C (control). Hydropriming consisted in soaking the seeds in distilled water at 20 °C for 3 h before transfer to the final temperature of 7 °C or 13 °C. This treatment was referenced to as ‘3 h’. The applied butenolide dilutions and duration of hydropriming were chosen based on the preliminary study.

### 4.3. Study Design

Seeds germinating in Petri dishes were divided into three groups. One day after sowing (das) a part of imbibed seeds was collected for analysis of cell membrane permeability expressed as electrolyte leakage (EL), dehydrogenase (DA) and α-amylase activity (A) as well as the content of non-active gibberellin GA_8_, active gibberellins (GAs), glucose conjugated abscisic acid (ABA-glc), free ABA, indole-3-acetic acid (IAA), kinetin (KIN) and 24-epibrassinolide (EPI). The second part of the seeds was left for evaluation of their germination vigour (4 das), while the third was sown (1 das) into boxes (V = 5 dm^3^; 20 seeds per box; three boxes per each combination of cultivar/temperature/treatment) filled with commercial soil (pH 6). The boxes were placed in growth chambers at 7 °C or 13 °C under light (photosynthetic active radiation—PAR) at 250 μmol·m^−2^·s^−1^. The plants were grown under 12 h/12 h photoperiod.

After two weeks (18 das), the percentage of germinated seeds in the soil was determined based on the number of seedlings. Four weeks after sowing, the vegetative phase developmental (VPD) was assessed and the plants grown under cold conditions were transferred to a chamber with temperature maintained at 13 °C where all studied plants were grown for the next four weeks. After this time (56 das), they were transferred to an open foil tunnel (50°06′97″ N, 19°84′57″ E), where they were cultivated from May until the end of July. Kinetics of chlorophyll *a* fluorescence and fresh (FW) and dry weight (DW) of the shoots were analysed in nine-week old plants. The effects of cold and seed treatment on the generative phase (GP) were evaluated 77 das. Successive collection of ripe seeds and evaluation of pod and seed number as well as seed weight began 19 weeks after sowing.

### 4.4. Measurements

#### 4.4.1. Determination of Seed Germination Vigour Index (Vi)

Germination vigour index was calculated according to the method described by Sparg et al. [35] with slight modifications. Germination vigour for each dish, estimated four days after sowing, was calculated based on hypocotyl length using the following visual scale: 1—hypocotyl length of 1 mm; 2—hypocotyl length of 2–3 mm; 3—hypocotyl length of 4–7 mm; and 4—hypocotyl length greater than 7 mm. The vigour (V) was calculated for each replicate (one dish) according to the formula:

VPD = [n_0_ × 1 + … + n_4_ × 4]/N (1)

where n_x_ is the number of seeds corresponding to a given hypocotyl length and N is the total number of seeds in a dish. The final number of germinated seeds was counted and expressed as percentage. The index of germination vigour was evaluated as:

Vi = V × percentage of germinated seeds
(2)

Analyses were performed in five replicates for each combination of cultivar/temperature/treatment.

#### 4.4.2. Electrolyte Leakage (EL)

Seeds collected one day after sowing were placed into vials containing 13 cm^3^ of ultrapure water (one seed per vial), and shaken (100 rpm) at 20 °C. After 24 h, electrical conductivity (E1) was measured using a conductometer (CI 317, Elmetron, Poland). The vials with samples were frozen for 24 h at −80 °C, and then thawed and shaken again. Conductivity measurements were repeated, and the resulting values represented total ion content (E2). Membrane permeability was expressed as the percentage of total EL according to the formula:

(E1 × 100/E2)
(3)

All measurements were performed in 10 replicates for each combination of cultivar/temperature/treatment.

#### 4.4.3. Dehydrogenase Activity Assay

The activity of dehydrogenase pool (DA) was measured according to Steponkus and Lanphear [40] with a slight modification. Seeds collected 1 das were weighed and placed into plastic vials (one seed per vial) along with 3 cm^3^ of a reaction mixture containing 1.5 cm^3^ of 0.4% (*v*/*w*) aqueous 2,3,5-triphenyltetrazolium chloride (TTC) and 1.5 cm^3^ of 0.1 M phosphate buffer (pH 7.5). The seeds were incubated for 3 h at 37 °C in darkness. Then they were homogenised with 5 cm^3^ of 96% ethanol to extract triphenylformazan, a product of TTC reduction by seed dehydrogenases. The extract was centrifuged at 16,000× *g* for 5 min. Finally, absorbance of the supernatant was measured at 485 nm using an Ultrospec 2100 Pro spectrophotometer (Amersham Biosciences, Umeå, Sweden). DA activity was expressed as μg of formazan per 1 g of protein. Protein concentration in seeds was determined spectrophotometrically according to Bradford [41] at 595 nm using the Bio-Rad (Munich, Germany) protein assay with bovine serum albumin as a standard. Analyses of DA activity and protein content were performed in five replicates for each combination of cultivar/temperature/treatment.

#### 4.4.4. α-Amylase (A) Activity Assay

Seeds collected one day after sowing were frozen with liquid nitrogen and stored at −80 °C. Prior to analysis, the seeds were grounded in mortars with 2 cm^3^ ice-cold 1M sodium phosphate buffer (pH 7.0), and then centrifuged (20,000× *g*) for 15 min. The supernatant was heated at 70 °C for 15 min in the presence of 1mM CaCl_2_ to inactivate β-amylase, debranching enzyme, and α-glucosidase [42]. Determination of α-amylase was carried out by quantifying the reducing sugar (maltose equivalent) liberated under the assay conditions. A modified dinitrosalicylic acid (DNS) method was adopted to estimate the maltose equivalent described by Bernfeld [43]. The heat-treated supernatant (125 μL) was added to 125 μL of 1% soluble starch in 100 mM Na-acetate buffer (pH = 4.5) containing 10 mM CaCl_2_. The mixture was incubated for 15 min at 37 °C and the reaction was stopped by adding 250 μL of DNS reagent (96 mM 3,5-dinitrosalicylic acid, 2 M NaOH, 1 M K-Na tartrate). After the addition of DNS, the samples were heated in boiling water bath for 5 min and then allowed to cool down on ice. For the blank sample, DNS was added prior to the substrate solution. Absorbance was measured at 540 nm. The reducing sugar formed by enzymatic activity was calculated using a standard curve for maltose. The results were expressed as U mg^-1^ protein. One unit (U) is defined as the amount of enzyme releasing 1 mg of maltose from starch in 15 min under the assay conditions. All measurements were performed in three replicates for each combination of cultivar/temperature/treatment.

#### 4.4.5. Hormone Content Analysis

Estimation of phytohormones (non-active GA_8_ and active GAs, ABA-glc and free ABA, IAA, KIN and EPI) was carried out as described by Hura et al. [44] with minor modifications. The seeds were freeze-dried and pulverised (MM400, Retch, Germany). The samples were spiked with stable isotope labelled internal standards ([^15^N_4_]kinetin, [^2^H_2_]gibberellin A1, [^2^H_2_]gibberellin A4, [^2^H_5_]indole-3-acetic acid, [^2^H_5_]dinor-12-oxo phytodienoic acid—internal standard for 24-epibrassinolide and [^2^H_6_]*cis*,*trans*-abscisic acid) and extracted. The extracts were cleaned up on SPE cartridges (Bond Elut Plexa PCX, 30 mg, 1 mm, Agilent, Santa Clara, CA, USA), and combined fractions of basic (cytokinin) and acidic (other) phytohormones after concentration under N_2_ were used for ultra-high performance liquid chromatography (UHPLC) analyses. The system consisted of UHPLC (Agilent Infinity 1260, Agilent, Waldbronn, Germany) and a triple quadruple mass spectrometer (Agilent 6410, Agilent, Santa Clara, CA, USA) with electro-spray ionization (ESI). Detailed measurement conditions were given by Dziurka et al. [45]. 

The monitored hormones ranked in elution order were: *Trans*-zeatin and *cis*-zeatin, [^15^N_4_]dihydrozeatin (used as internal standard, ISTD), [^15^N_4_]kinetin (ISTD) and kinetin, gibberellin A_8_ (GA_8_), [^2^H_5_]trans-zeatin riboside (ISTD), *trans*-zeatin riboside, and *cis*-zeatin riboside, ^6^N-izopentenyladenine, kinetin riboside, [^2^H_5_]indole-3-acetic acid (ISTD) and indole-3-acetic acid (IAA), gibberellic acid (GA_3_), [^2^H_2_]gibberellin A_1_ (ISTD), gibberellin A_1_ (GA_1_), abscisic acid glucosyl ester (ABA-glc), gibberellin A_6_ (GA_6_), [^2^H_6_]*cis*,*trans*-abscisic acid (ISTD), *cis*,*trans*-abscisic acid (ABA), gibberellin A_5_ (GA_5_), [^2^H_2_]gibberellin A_4_, gibberellin A_4_ (GA_4_), [^2^H_5_]dinor-12-oxo phytodienoic acid (ISTD) and 24-epibrassinolide (EPI). Multiple reactions monitoring (MRM) transitions were used for identification and quantification of all compounds of interest (details in Appendix A, Table A1). MassHunter software was used to control the LC–MS/MS system and in data analysis. For MRM parameters optimization MassHunter Optimizer was used. Calibration was based on data obtained for pure standards in conditions identical like for samples, taking in to account recoveries of ISTD.

A standard of [^2^H_5_]dinor-12-oxo phytodienoic acid was purchased from Cayman Chemical Company (Michigan, MI, USA), whereas all other reagents were from OlChemIm (Olomouc, Czech Republic) and were of the highest available purity. The data were presented as fmol (femtomol)·g^−1^ DW. Analyses were performed in three replicates for each combination of cultivar/temperature/treatment.

#### 4.4.6. Visual Analysis of Plant Vegetative Phase Development (VPD)

Vegetative phase was evaluated 28 das using the following visual scale 1–4, where: 1—only cotyledons; 2—the first leaves begin to appear; 3—small leaves of the first order; 4—fully developed leaves of the first order. The VPD coefficient was calculated as:

VPD = [n_0_ × 1 + … + n_4_ × 4]/N (4)

where n_x_ is the number of plants corresponding to a given developmental phase and N is the total number of plants in a box. Index of VPD was calculated as:

VPDi = VPD × percentage of germinated seeds in soil
(5)

One box was one replicate, while the analysis was performed in three replicates for each combination of cultivar/temperature/treatment.

#### 4.4.7. Determination of Fresh and Dry Weight

Fresh (FW) and dry weight (DW) of the shoots was evaluated in nine-week old plants. The shoots were cut (10 plants per cultivar, temperature and treatment), weighed to determine the FW and then dried at 70 °C for 48 h to determine the DW.

#### 4.4.8. Measurements of PSII Efficiency (Kinetics of Chlorophyll *a* Fluorescence)

PSII efficiency was measured using a Plant Efficiency Analyzer (PEA; Hansatech Ltd., King’s Lynn, Norfolk, UK) with an excitation light intensity of 3 mmol·m^−2^·s^−1^ (peak 650 nm). The measurements were carried out in fully developed leaves of the third order after 30 min of adaptation to darkness in leaf clips (Hansatech, Norfolk, UK). The following parameters of PSII efficiency including phenomenological fluxes and activities were calculated based on Strasser et al. [46]: ABS/CSm—energy absorption by antennas; TRo/CSm—amount of excitation energy trapped in PS II (energy transferred to a reaction centre); ETo/CSm—amount of energy used for electron transport; DIo/CSm—energy dissipation from PSII (energy lost as heat), where CSm is the cross section of a sample; PI—performance index of PSII normalised for equal absorption (PIABS); RC/CSm—number of active reaction centres. The measurements were performed on nine-week old plants, ten plants per each combination of cultivar/temperature/treatment (10 replicates).

#### 4.4.9. Visual Analysis of Plant Generative Development (GD)

Generative development was assessed with the following 0–2 scale, where: 0—only vegetative phase; 1—first flower bud; 2—appearance of flowers in the inflorescence. The GD was calculated similarly as DVP. Analysis was carried out on 11-week old plants, 10 plants per each combination of cultivar/treatment/temperature.

#### 4.4.10. Analysis of Yield Parameters

Due to asynchronous ripening, the seeds were collected successively 19 weeks after sowing. The number of pods, seeds, and seed weight were calculated per plant. Analysis was performed in 10 replicates (10 plants) per each combination of cultivar/temperature/treatment.

#### 4.4.11. Statistical Analysis

The experiments were arranged in a fully randomised design. Analysis of variance was performed using the statistical package STATISTICA 12.0 (Stat-Soft, Inc., Tulsa, OK, USA). Linear correlation coefficients (Pearson’s) were assumed as statistically significant at p < 0.05. All data were presented as means ±SE.

## 5. Conclusions

Narrow-leaf lupine seeds that germinate well at low temperature are characterised by higher α-amylase activity, greater amount of gibberellins, IAA, and kinetin, when compared with weakly germinating seeds. Germination ability at 7 °C correlates positively with dehydrogenase activity and negatively with cell membrane permeability. It is difficult to determine the concentration of butenolide most strongly affecting the studied parameters. However, more pronounced results are observed after seed treatment with butenolide of lower concentration i.e., 10^−6^ M. Seed hydropriming for 3 h at 20 °C and butenolide applied before sowing, improve germination at low temperature by a reduction of ABA levels as compared with control seeds germinating in water. Butenolide alleviates the effects of cold during seed germination, while 3-h hydropriming is more effective at later growth phases when it accelerates vegetative and generative development. Butenolide at the concentrations of 10^−6^ M to 10^−4^ M increases the activity of dehydrogenases and declines the amount of inactive GA_8_ in germinating seeds. Hydropriming increases the amount of active gibberellins, especially at low temperature. Germination vigour of seeds correlates with plant fresh and dry weight as well as with the number of pods, so it should be assumed that the seed germination vigour might be used to predict potential seed yield. Moreover, the overall efficiency of photosynthesis measured before flowering could be used to predict the yield of narrow-leaf lupine.

## Figures and Tables

**Table 1:** Effects of temperature and seed treatment (two-way ANOVA) on germination vigour index (Vi), ion leakage (EL), dehydrogenase (DA) and α-amylase (A) activity, content of inactive gibberellin (GA_8_), active gibberellins (GAs), conjugated abscisic acid (ABA-glc), free ABA, kinetin (KIN), indole-3-acetic acid (IAA) and 24-epibrassinolide (EPI) in the seeds of two *L. angustifolius* cultivars germinated at 7 °C or 13 °C in water or after pre-sowing treatments: Three-hour hydropriming or 10^−6^ M, 10^−5^ M, 10^−4^ M butenolide.

Source of Variance	df	Vi	EL	DA	A	GA8	GAs	ABA-Glu	Free ABA	KIN	IAA	EPI
Cultivar (C)	1	*	***	*	*	***	***	**	**	***	*	*
Temperature (t)	1	**	*	**	ns	***	***	*	*	**	**	*
Treatment (T)	4	*	ns	***	ns	*	*	*	*	*	ns	*
C × t	1	ns	ns	ns	ns	**	**	ns	ns	ns	ns	ns
C × T	1	ns	ns	ns	ns	**	**	ns	ns	ns	ns	ns
C × t × T	4	ns	ns	ns	ns	ns	ns	ns	ns	ns	ns	ns

*, **, ***, indicate statistically significant effect of temperature at *p* < 0.05, *p* < 0.01, *p* < 0.001, respectively, ns—not significant.

**Table 2:** Effects of temperature and pre-sowing seed treatment (two-way ANOVA) on vegetative phase development index (VPDi), fresh (FW) and dry weight (DW) of shoots and generative development (GD) of two *L. angustifolius* cultivars grown for the first four weeks at 7 °C or 13 °C from seeds three-hour hydroprimed or treated with 10^−6^ M, 10^−5^ M or 10^−4^ M butenolide.

Source of Variance	df	VPDi	FW	DW	GD
Cultivar (C)	1	**	ns	ns	***
Temperature (t)	1	***	***	***	**
Treatment (T)	4	**	*	ns	**
C × t	1	*	**	**	*
C × T	1	*	*	*	*
C × t × T	4	ns	*	*	*

*, **, ***, indicate statistically significant effect of treatment at *p* < 0.05, *p* < 0.01, *p* < 0.001, respectively, ns—not significant.

**Table 3:** Effects of pre-sowing treatments on the index of vegetative phase development (VPDi) of two *L. angustifolius* cultivars germinated and grown for the first four weeks at 7 °C or 13 °C. Control—plants grown from seeds germinated in water; 3 h—plants grown from seeds hydroprimed for 3 h at 20 °C; But 1, But 2, But 3—plants grown form seeds germinated in the presence of 10^−6^ M, 10^−5^ M, 10^−4^ M butenolide, respectively.

Temperature (°C)	Treatment	Lazur	Sethes Fruehe Rote
7	Control	251 ± 22c	138 ± 11f
	3 h	250 ± 21c	142 ± 12ef
	But 1	141 ± 15e	72 ± 6g
	But 2	150 ± 16e	52 ± 5h
	But 3	190 ± 20d	156 ± 17e
13	Control	356 ± 32a	252 ± 23c
	3 h	360 ± 33a	208 ± 19d
	But 1	352 ± 31a	311 ± 29b
	But 2	312 ± 29b	413 ± 39a
	But 3	324 ± 30ab	308 ± 27b

Values represent means (*n* = 3) ±SE. Different superscript letters (a, b, c, …) in the columns indicate significant differences between means (Duncan’s multiple range test, *p* < 0.05). VPD was evaluated using visual scale 1–4 where: 1—only cotyledons; 2—the first leaves begin to appear; 3—small leaves of the first order; 4—fully developed leaves of the first order. VPD was calculated as: VPD = [n_0_ × 1 + … + n_4_ × 4]/N, where n_x_ is the number of plants corresponding to a given developmental phase and N is the total number of plants in a box. Index of VPD was calculated as: VPDi = VPD × percentage of germinated seeds in the soil.

**Table 4:** Effects of pre-sowing treatments on fresh (FW) and dry weight (DW) [g] of two *L. angustifolius* cultivars germinated and grown for the first four weeks at 7 °C or 13 °C. Control—plants grown from seeds germinated in water; 3 h—plants grown from seeds hydroprimed for 3 h at 20 °C; But 1, But 2, But 3—plants grown form seeds germinated in the presence of 10^−6^ M, 10^−5^ M, 10^−4^ M butenolide, respectively.

Temperature (°C)	Treatment	Lazur		Sethes Fruehe Rote	
		FW	DW	FW	DW
7	Control	3.54 ± 0.42d	0.559 ± 0.071e	5.15 ± 0.61b	0.748 ± 0.121c
	3 h	4.31 ± 0.51c	0.691 ± 0.093cd	4.90 ± 0.58bc	0.757 ± 0.122c
	But 1	3.65 ± 0.43d	0.575 ± 0.072e	4.59 ± 0.60c	0.599 ± 0.077d
	But 2	4.34 ± 0.52c	0.714 ± 0.093bc	3.10 ± 0.37e	0.612 ± 0.073d
	But 3	3.33 ± 0.40d	0.546 ± 0.071e	3.55 ± 0.42d	0.591 ± 0.071d
13	Control	4.48 ± 0.49c	0.774 ± 0.100b	7.09 ± 0.85a	1.016 ± 0.122a
	3 h	5.91 ± 0.70a	0.934 ± 0.112a	4.11 ± 0.50c	0.959 ± 0.116ab
	But 1	4.71 ± 0.52c	0.774 ± 0.100b	4.52 ± 0.54c	1.006 ± 0.141a
	But 2	5.22 ± 0.63b	0.850 ± 0.110ab	3.86 ± 0.46d	1.006 ± 0.141a
	But 3	3.93 ± 0.43d	0.605 ± 0.071d	5.56 ± 0.67b	0.877 ± 0.123b

Values represent means (*n* = 20) ±SE. Different superscript letters (a, b, c, …) in the columns indicate significant differences between means (Duncan’s multiple range test, *p* < 0.05). Analysis was done after 63 days.

**Table 5:** Effects of temperature and pre-sowing seed treatment (two-way ANOVA) on the kinetics of chlorophyll *a* fluorescence in plants of two *L. angustifolius* cultivars grown at 7 °C and 13 °C for the first four weeks from seeds germinated in water, three-hour hydroprimed or treated with 10^−6^ M, 10^−5^ M, 10^−4^ M butenolide.

Source of Variance	df	ABS/CSm	TRo/CSm	ETo/CSm	DIo/CSm	RC/CSm	PI
Cultivar (C)	1	**	***	**	**	ns	***
Temperature (t)	1	**	***	***	*	ns	***
Treatment (T)	4	ns	ns	ns	*	*	**
C × t	1	ns	*	ns	ns	*	**
C × T	1	ns	ns	*	ns	**	ns
C × t × T	4	*	**	ns	*	**	**

*, **, ***, indicate statistically significant effect of treatment at *p* < 0.05, *p* < 0.01, *p* < 0.001, respectively, ns—not significant.

**Table 6:** Effects of pre-sowing treatments on generative development (GD) of two *L. angustifolius* cultivars germinated and grown for the first four week at 7 °C or 13 °C. Control—plants grown from seeds germinated in water; 3 h—plants grown from seeds hydroprimed for 3 h at 20 °C; But 1, But 2, But 3—plants grown form seeds germinated in the presence of 10^−6^ M, 10^−5^ M, 10^−4^ M butenolide, respectively.

Temperature (°C)	Treatment	Lazur	Sethes Fruehe Rote
7	Control	1.79 ± 0.13c	0.69 ± 0.05b
	3 h	2.00 ± 0.16a	0.77 ± 0.06b
	But 1	1.58 ± 0.12d	0.31 ± 0.02c
	But 2	1.50 ± 0.12d	0.38 ± 0.03c
	But 3	1.48 ± 0.11d	0.06 ± 0.01d
13	Control	1.83 ± 0.15bc	0.80 ± 0.07a
	3 h	2.00 ± 0.16a	0.90 ± 0.08a
	But 1	1.50 ± 0.12d	0.83 ± 0.07a
	But 2	1.92 ± 0.14ab	0.67 ± 0.05b
	But 3	1.75 ± 0.14c	0.33 ± 0.03c

Values represent means (*n* = 10) ±SE. Different superscript letters (a, b, c, …) in the columns indicate significant differences between means (Duncan’s multiple range test, *p* < 0.05). Analysis was done 77 das using a visual scale 0–2 where: 0—only vegetative phase; 1—first flower bud; 2—appearance of flowers in the inflorescence.

**Table 7:** Effects of temperature and pre-sowing seed treatment on the number of pods, seeds and seed weight per plant of two *L. angustifolius* cultivars grown for the first four weeks at 7 °C or 13 °C from seeds germinated in water, three-hour hydroprimed or treated with 10^−6^ M, 10^−5^ M, 10^−4^ M butenolide.

Source of Variance	df	No. of Pods	No. of Seeds	Seed Weight
Cultivar (C)	1	***	***	*
Temperature (t)	1	***	*	ns
Treatment (T)	4	***	***	***
C × t	1	***	***	*
C × T	1	**	***	***
C × t × T	4	*	***	***

*, **, ***, indicate statistically significant effect of treatment at *p* < 0.05, *p* < 0.01, *p* < 0.001, respectively, ns—not significant.

**Table 8:** Effects of pre-sowing treatments on number of pods, seeds and seed weight (g) per plant of two *L. angustifolius* cultivars germinated and grown for the first 4 weeks at 7 °C (cold) or 13 °C (control temperature). Control—plants grown from seeds germinated in water; 3 h—plants grown from seeds hydroprimed for 3 h at 20 °C; But 1, But 2, But 3—plants grown form seeds germinated in 10^−6^ M, 10^−5^ M, 10^−4^ M butenolide, respectively.

Temperature (°C)	Treatment	Lazur			Sethes Fruehe Rote		
		No. of Pods	No. of Seeds	Seed Weight	No. of Pods	No. of Seeds	Seed Weight
7	Control	6.3 ± 0.4a	15.7 ± 1.2b	1.28 ± 0.05c	3.3 ± 0.2b	12.9 ± 0.9bc	1.89 ± 0.17b
	3 h	6.3 ± 0.6a	18.6 ± 1.0a	1.69 ± 0.16ab	3.2 ± 0.3b	13.4 ± 1.2b	1.91 ± 0.14b
	But 1	5.1 ± 0.4bc	13.8 ± 1.0c	1.21 ± 0.09c	3.6 ± 0.5b	13.3 ± 2.0b	1.77 ± 0.28bc
	But 2	5.3 ± 0.7bc	19.0 ± 2.3a	1.74 ± 0.27a	3.1 ± 0.4b	9.1 ± 0.9d	1.31 ± 0.15e
	But 3	4.8 ± 0.4c	13.2 ± 1.2c	1.15 ± 0.07d	1.7 ± 0.2d	5.6 ± 0.6e	0.72 ± 0.11g
13	Control	5.8 ± 0.4ab	11.8 ± 1.4d	1.29 ± 0.13c	3.6 ± 0.4b	12.2 ± 1.5c	1.69 ± 0.32c
	3 h	6.6 ± 0.7a	15.8 ± 1.3b	1.55 ± 0.10b	4.7 ± 0.3a	17.0 ± 1.4a	2.28 ± 0.29a
	But 1	3.9 ± 0.6d	9.0 ± 1.4d	0.86 ± 0.13e	3.0 ± 0.2b	11.0 ± 0.6c	1.49 ± 0.08d
	But 2	6.1 ± 0.5a	13.9 ± 0.9c	1.48 ± 0.12b	2.7 ± 0.2c	9.6 ± 0.8d	1.17 ± 0.12f
	But 3	5.2 ± 0.3bc	14.2 ± 1.7bc	1.29 ± 0.17c	2.6 ± 0.2c	9.2 ± 1.0d	0.77 ± 0.12g

Values represent means (*n* = 10) ±SE. Different superscript letters (a, b, c, …) in the columns indicate significant differences between means (Duncan’s multiple range test, *p* < 0.05).

## Abbreviations

3 h	seed hydropriming at 20 °C for 3 h
A	α-amylase activity
ABA	abscisic acid
ABA-glc	glucose conjugated abscisic acid
ABS/CSm	energy absorption by antennas
ANOVA	analysis of variance
butenolide	2(5*H*)-furanone
But 1	seed treatment with 10^−6^ M butenolide
But 2	seed treatment with 10^−5^ M butenolide
But 3	seed treatment with 10^−4^ M butenolide
DA	dehydrogenase activity
das	days after sowing
DIo/CSm	energy dissipation from PSII (energy lost as heat), where CSm is the cross section of the sample
DW	dry weight
EL	electrolyte leakage
ETo/CSm	energy used for electron transport
FW	fresh weigh
IAA	indole-3-acetic acid
KIN	kinetin
PAR	photosynthetic active radiation
PI	performance index of PSII normalised for equal absorption (PI/ABS)
PSII	photosystem II
Q_A_	primary quinone acceptor of PSII
RC/CSm	number of active reaction centres
TRo/CSm	excitation energy trapped in PSII (energy transferred to a reaction centre)
VPD	coefficient of the vegetative phase development
VPDi	index of the vegetative phase development

## Appendix A

**Table A1:** Multiple reactions monitoring (MRM) transitions for the analysed phytohormones at: Positive ion mode (+ESI), capillary voltage 4 kV, gas temperature 350 °C, gas flow 12 L/min and nebuliser pressure 35 psi.

Compound	Type of Ion	Quantifier Transition (Precursor/Product Ions)	Fragmentor Voltage (V)	Collision Energy (V)	MRM Start Time (min)
*trans*-zeatin	[M + H]^+^	220.2/136.3	85	9	1.6
*cis*-zeatin	[M + H]^+^	220.2/136.3	85	9	
[^15^N_4_]dihydrozeatin	[M + H]^+^	226.2/140	124	18	
[^15^N_4_]kinetin	[M + H]^+^	220.1/192.3	90	9	4.0
kinetin	[M + H]^+^	216.1/188.3	90	9	
GA8	[M-H_2_O + H]^+^	319.3/257.2	102	9	
[^2^H_5_]trans-zeatin riboside	[M + H]^+^	357.3/225.2	116	17	
*trans*-zeatin riboside	[M + H]^+^	352.2/220.3	120	9	
*cis*-zeatin riboside	[M + H]^+^	352.2/220.3	120	9	
^6^N-izopentenyladenine	[M + H]^+^	204.1/148.3	90	9	7.4
kinetin riboside	[M + H]^+^	348.2/216.3	116	9	8.5
[^2^H_5_]indole-3-acetic acid	[M + H]+	181.1/135.1	38	14	
IAA	[M + H]+	176.1/130.3	51	9	
GA3	[M-H_2_O + H]^+^	329.3/311.3	100	14	
[^2^H_2_]gibberellin A_1_	[M-H_2_O + H]^+^	333.3/287.2	58	9	10.3
GA_1_	[M-H_2_O + H]^+^	331.3/285.3	100	14	
ABA-glc	[M-H_2_O + H]^+^	409.2/247.1	104	14	
GA_6_	[M-H_2_O + H]^+^	329.3/283.3	104	14	12.4
[^2^H_6_]*cis*,*trans*-abscisic acid	[M-H_2_O + H]^+^	253.4/191.3	80	14	16.7
ABA	[M-H_2_O + H]^+^	247.4/187.2	80	14	
GA_5_	[M-H_2_O + H]^+^	285.1/267.1	96	5	
[^2^H_2_]gibberellin A_4_	[M-H_2_O + H]^+^	317.3/271.2	88	9	20.9
GA_4_	[M-H_2_O + H]^+^	315.3/269.3	100	14	
EPI	[M + H]^+^	481.4/445.3	112	9	23.1
[^2^H_5_]dinor-12-oxo phytodienoic acid	[M + H]^+^	270.3/252.2	84	5	
